# The use of Schirmer strips to measure salivary and lacrimal flow in non-Sjögren patients

**DOI:** 10.1007/s00784-020-03741-3

**Published:** 2021-01-03

**Authors:** Dominika Wróbel-Dudzińska, Agnieszka Kubik-Komar, Dorota Rykwa, Ewa Kosior-Jarecka, Tomasz Żarnowski, Renata Chałas

**Affiliations:** 1 Department of Diagnostics and Microsurgery of Glaucoma, Medical University of Lublin, Ul Chmielna1, 20-079 Lublin, Poland; 2 Department of Applied Mathematics and Computer Science, University of Life Sciences in Lublin, Lublin, Poland; 3 Department of Oral Medicine, Medical University of Lublin, Lublin, Poland

**Keywords:** Schirmer test, Non-Sjögren patients, Dry eye, Xerostomia

## Abstract

**Introduction:**

Oral and ocular dryness are the most common symptoms reported during ophthalmological and dental examinations. It is becoming a serious and growing problem due to the huge variety of affecting factors and with population aging.

**Objectives:**

The purpose was to demonstrate an application of the Schirmer test for xerostomia. Subsequently, to compare and correlate the results achieved from the lacrimal Schirmer test and salivary Schirmer test in non-Sjögren patients.

**Methods:**

Study group consisted of 642 patients with/without subjective or/and objective symptoms of dry eye or mouth who did not fulfill the criteria for diagnosis of Sjögren syndrome. The lacrimal Schirmer test (lST) and the salivary Schirmer tests (sST) were performed (sSTm was put on the floor of the mouth, sSTp in front of the parotid gland duct). The results were recorded after 1 min (sSTm), 3 min (sSTp), and 5 min (lST).

**Results:**

The lST and sST test scores were considerably higher in the healthy group than in others, *p* < 0.001. The results of sST1 and sST2 decreased with the appearance of subjective and objective symptoms, *p* < 0.001. There were positive correlations between lST and sSTm outcomes between the groups, *p* < 0.001.

**Conclusions:**

We present the Schirmer test adapted to measure salivary gland hypofunction that is a time-saving tool in our daily practice. Results of this study reveal an excellent correlation between the eye Schirmer test and the salivary Schirmer tests.

**Clinical relevance:**

The salivary Schirmer tests seem to be rapid, convenient, and reliable objective screening tools for salivary gland hypofunction in non-Sjögren patients.

## Introduction

The prevalence of xerostomia, defined as the subjective sensation of dry mouth [1], ranges from 20 to 80% of the population [2]. Salivary gland hypofunction may be a symptom of a serious systemic disease and may be related to the use of various drugs. Many widely known techniques used for an estimation of xerostomia are based on the measurement of salivary flow rate and are therefore resisted on stimulators like paraffin, chewing gum, or Saxon [3]. Moreover, they are time-consuming, unpleasant for the patients, and thus reluctantly used. There are still no objective and direct measurement methods to evaluate the resting moisture of the oral cavity.

The keratoconjunctivitis sicca is one of the most prevalent conditions in ophthalmological practice, occurring in 5–50% of general population [4]. In 1903, Otto Schirmer devised a tear test using a Whatman (number 41) special filter paper strip of 35 length and 5 mm width [5] that was hooked over the margin of the lower lid (without previous administration of anesthetic) with closed eyes. It was maintained for 5 min to measure basal tear secretion [6]. According to the literature, wetting of less than 5 or 10 mm/5 min without anesthesia is indicative of aqueous tear deficiency and is a classification criterion for Sjögren syndrome [7, 8].

We present an application of the Schirmer test for salivary gland hypofunction, compare, and correlate results obtained from the lacrimal and the salivary Schirmer tests between non-Sjögren patients with the healthy control group.

## Materials and methods

### Study population

Undertaken investigation was an observational pilot study, not randomized and not prospective. The research was conducted in the years 2011–2015 on 642 adults from the Lublin district area. They were referred to ophthalmologist or dentist because of their symptoms, or for preventive examinations (due to the nature of the done work or the driving license test), some patients due to diabetes or hypertension. All patients gave written informed consent to participate in the study. The Bioethics Committee consent was obtained from the Medical University of Lublin, Poland (No. KE-0254/227/2014). The investigation was conducted in compliance with the tenets of the Declaration of Helsinki and Polish regulations.

### Exclusion and inclusion criteria

#### Exclusion criteria

Patients, above 18 years of age, suffering from Sjögren’s syndrome, multiple systemic diseases, and craniofacial injuries, and those undergoing head and neck irradiation therapy were excluded. High refractive errors, acute inflammation in the oral cavity and the eye, history of allergic conjunctivitis, lid abnormalities, glaucoma, pterygium, and extra- and intraocular surgery within the last 6 months were exclusion criteria. Patients who wore contact lenses; smoked; took eye or dental anti-inflammatory drugs (antibiotics, steroids) during the month before visit; and took local or systemic medications known to cause dry eyes and mouth like antihistaminic, anticholinergic, antidepressants, antipsychotics, bronchodilators, and chemiotherapeutic agents were also excluded from the study.

#### Inclusion criteria

Patients who did not meet exclusion criteria were selected for the study and underwent a comprehensive ophthalmological and oral cavity examination. Moreover, the study used the Interview and Examination Questionnaire to gauge lifestyle, with a special emphasis on computer work (more than 8 h per day); staying in polluted and air-conditioned rooms (more than 8 h per day); addictions; and past and existing ophthalmic, dental, and systemic diseases. Subsequent questions concerned the subjective symptoms of the mouth and eye (burning sensation of the eyes, itching of the eyes, sand under the eyelids, blurred vision, need to rub or compression of the eyelids, dryness at night and during the day and meal, difficulty in swallowing, burning in the mouth or the tongue, halitosis, necessity of moisturizing during the day and the night, necessity of chewing gum or candies). The questions were closed single choice.

Patients were divided on the basis of history and examination, into the following groups:

A.One hundred fifty-six generally healthy subjects, asymptomatic (either eye or mouth), absence of clinical symptoms,

B.One hundred nine generally healthy cases, asymptomatic (either eye or mouth), with clinical symptoms of dryness from eye or mouth,

C.One hundred three generally healthy people with subjective and clinical symptoms of dryness from eye or mouth,

D.One hundred fifteen patients with systemic diseases, asymptomatic (either eye or mouth) without clinical symptoms,

E.Eighty-four subjects with systemic diseases, asymptomatic (either eye or mouth) with clinical symptoms of dryness from eye or mouth,

F.Seventy-five cases with systemic diseases, and subjective and clinical symptoms of dryness from eye or mouth.

### Ophthalmological and dental examination

The eye and dental examinations were concentrated on clinical symptoms: corneal epithelial defects, lack of tear meniscus, conjunctival folds (LIPCOF I–IV), erosions in the oral cavity, mirror test II–III). The mirror test, used to assess the severity of dry mouth, is based on the assessment of the movement of the dental intraoral mirror along the buccal mucosa or tongue. The results are presented in a three-item scale: (I) a lack of resistance during the passing of a dental mirror along the oral mucosa; (I) slight resistance noticeable; (III) significant resistance during an attempt to pass the mirror, the mirror sticks to the film mucous.

During ophthalmology examination, slit lamp and fluorescein dye were used. Then, the dental mirror was applied to the surface of the oral cavity mucosa and gently moved. Degrees II and III corresponded to slight or significant resistance when moving the dental mirror over the surface of the oral mucosa.

### Schirmer tests (lacrimal and salivary Schirmer tests)

**Fig. 1:**
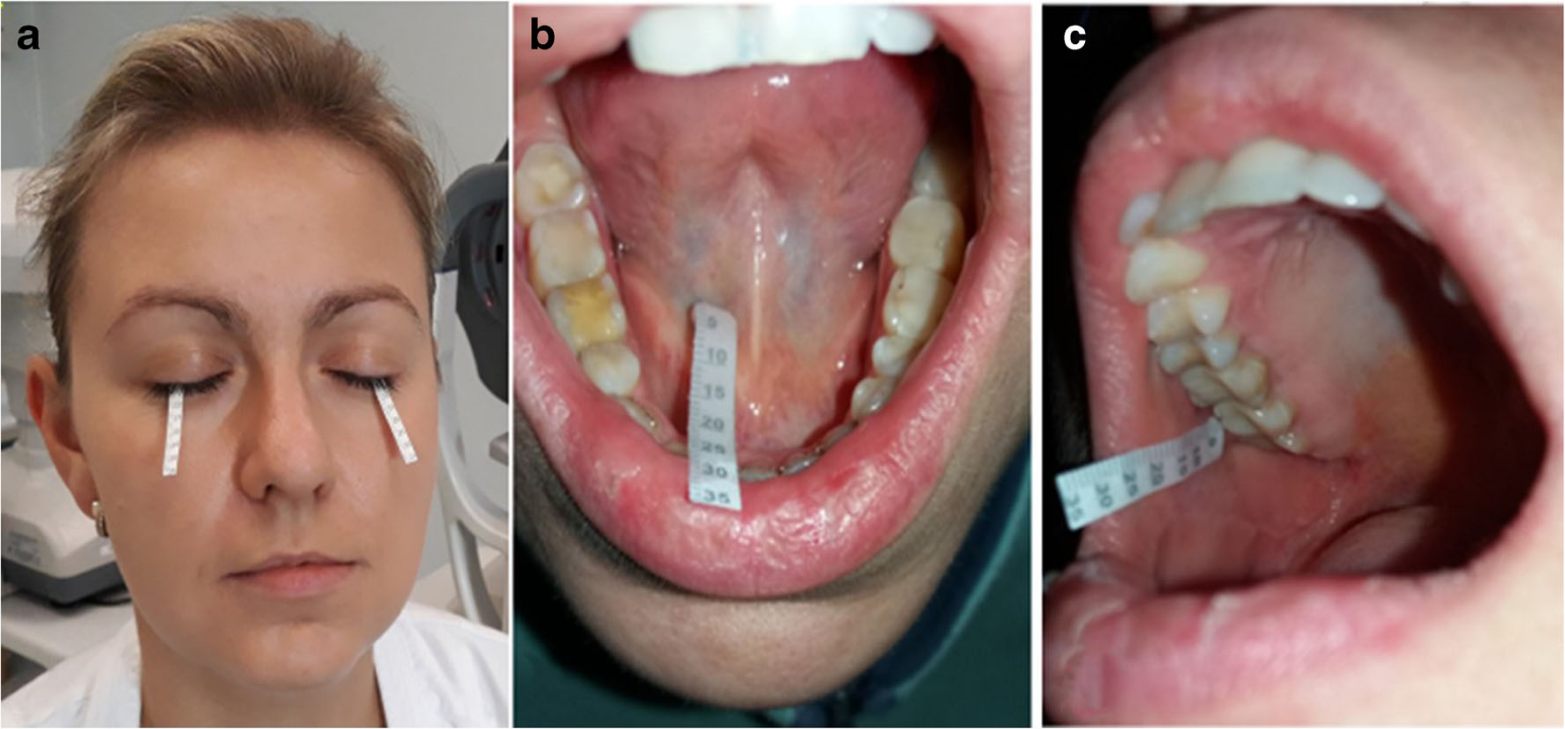
**a** Lacrimal Schirmer test, **b** Salivary Schirmer test 1. **c** Salivary Schirmer test 2

The tear secretion was measured by the Schirmer I test *without previous* instillation of topical anesthetic (unstimulated test). Standardized strips (a Whatman (number 41) special filter paper strip of 35 length and 5 mm width) were placed in the lateral third of the lower eyelid, and the length of the moistened portion of the strip after 5 mins with closed eyes was measured. It was maintained to measure basal and reflex secretion of tears (Fig. 1a). Salivary unstimulated Schirmer tests (sST1 and sST2) were performed in the morning to measure an unstimulated salivary flow. Patients were not allowed to eat, drink, smoke, or brush their teeth 2 h before the examination. Participants were asked to sit upright in a chair, swallow all the saliva in the mouth before the test, and not swallow any more during the tests. Moreover, patients were requested to open their mouths and to rest their tongue on the hard palate and to prevent inadvertent wetting of the test strips. When the sSTm test was performed, the strip was placed in the floor of the mouth, next to the submandibular gland duct/the lingual frenum (Fig. 1b). During the sSTp, the strip was put in front of the parotid gland duct (Fig. 1c). Based on the length of wetting, the results were recorded after 1-min (sSTm), 3-min (sSTp), and 5-min intervals (lST).

### Statistical analysis

Statistical analyses were performed using the Statistica 12.0 PL software (StatSoft Poland, Cracow). The Shapiro-Wilk test was used to test the distribution of variables. The results were reported mainly as mean value (M) ± standard deviation (SD) or percentage values. A *p* value of less than 0.001 was considered statistically significant due to the large number of the study group. A one-way ANOVA analysis of variance with Tuckey post hoc test analysis was performed to compare the means between groups. Multi-factor analysis of variance (ANOVA) was used to measure the differences between groups. Repeated measures ANOVA test with Bonferroni’s correction for multiple comparisons was performed to assess the changes of variables over time. Categorical variables were compared using the chi-squared test. To compare relationships between variables, parametric testing (Pearson correlation test) was used.

## Results

### Demographic and clinical characteristics

The demographic data including age, number of patients in each group, and results of the lacrimal Schirmer test and the salivary Schirmer tests are presented in Tables 1 and 2. The group of healthy patients was the youngest, while participants with general diseases, and subjective and clinical symptoms of eye and mouth dryness were the oldest (*p* < 0.001). There were also significant differences in ages between group A and others, A vs B *p* = 0.064; A vs C *p* = 0.006; and A vs D, E, and F *p* < 0.001. The female gender was more common among the study groups 63.08% (405 female) vs 36.92% (237 male), *p* = 0.043.

**Table 1:** Demographic characteristics

Group	A	B	C	D	E	F	Mean
No.	156	109	103	115	84	75	642
Age M ± SD	45.1 ± 12.2	49.0 ± 11.1	50.0 ± 10.7	52.3 ± 8.4	51.7 ± 9.2	57.7 ± 12.1	50.2 ± 11.4
Gender M:F	64:92	42:67	17:86	37:78	39:45	38:37	237:405

**Table 2:** Schirmer test results

Group	Salivary Schirmer test mouth (after 1 min)		Salivary Schirmer test parotid (after 1 min)		Lacrimal Schirmer test (after 5 min)		ANOVA	
	*M*	*SD*	*M*	*SD*	*M*	*SD*	*F*	*p*
Mean	14.51	3.09	20.33	4.82	14.28	3.34	5237.35	< 0.001
A	18.56	1.79	26.85	2.81	18.61	2.23	1888.45	< 0.001
B	14.31	1.48	20.16	2.33	14.53	1.87	1127.28	< 0.001
C	12.34	1.83	16.61	2.39	12.47	1.65	1058.99	< 0.001
D	15.34	1.56	21.57	1.96	14.68	1.66	2725.25	< 0.001
E	12.47	1.83	17.41	2.71	12.39	1.70	1235.88	< 0.001
F	11.00	1.79	14.5	2.38	9.55	1.29	1004.76	< 0.001

### Systemic disease distribution

Medical history taken in the study group revealed systemic diseases such as hypertension (32.87%), diabetes mellitus (7.63%), cardiovascular disease (8.41%), and hyperthyroidism (3.27%).

### Subjective and objective symptoms in the study groups

According to our survey results, participants complained of subjective eye symptoms such as foreign body sensation (26%), itching (16.82%), and burning eyes (18.32%). They complained of mouth symptoms such as mouth dryness (35.82%), burning sensation (23.36%), the need for moisturizing the lips (31%), or eating sweets and chewing gum (6.07%) to stimulate saliva production. The most common objective symptoms of the ocular surface and mouth dryness were conjunctival folds (52.96%) (LIPCOF I—20.09%, II—21.5%, III—8.41%, IV—2.96%), lack of tear meniscus (26.64%), corneal epithelial defects (2.96%), mirror test (31.31%) (II—26.64%, III—4.67%), and erosions in the oral cavity (3.11%). The sicca symptoms correlated with values for unstimulated saliva flow and tear flow.

### Lacrimal and salivary flow rate tests

**Fig. 2:**
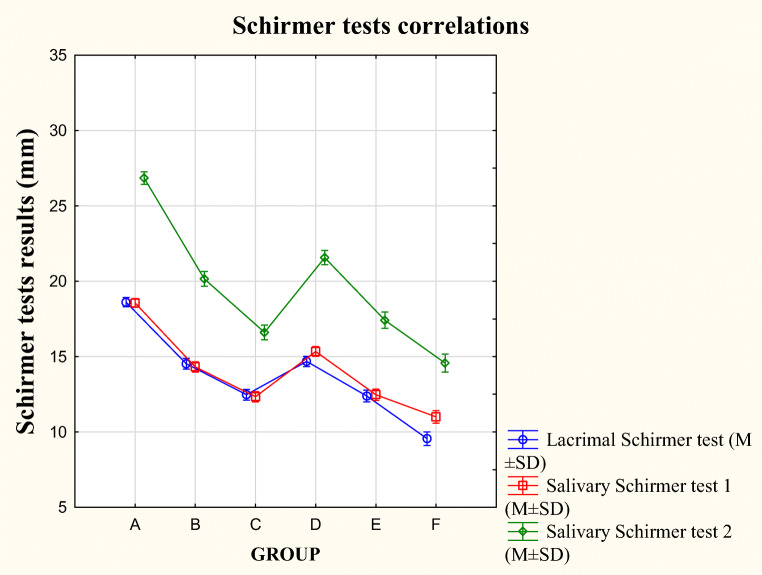
The lST, sSTm, and sSTp results in the studied groups

The lST results were significantly higher in the healthy patients (group A) when compared to the other groups, *p* < 0.001. Interestingly, in groups C and F, none of the patients achieved normal tear production results. The lST results were higher in the healthy patients (group A), and significant differences were reached when compared to other groups (A vs B, C, D, E, and F, *p* < 0.001) (Table 2, Fig. 2). Moreover, differences were found between groups B vs E and C vs F, *p* < 0.001.

The mean strip wetness at the end of first minute for sSTm was 14.51 mm ± 3.09, respectively for sSTp 20.33 mm ± 4.82. Significant difference existed between the study groups. None of the patients from group B to F achieved sSTm above 21 mm (A vs B–F, *p* < 0.001). There were large differences observed in the obtained outcomes of the sSTm and 2 between the study groups, *p* < 0.001. Similar to the eye Schirmer test, the results of sSTm and sSTp decreased with the appearance of subjective and objective symptoms, *p* < 0.001. A significantly lower unstimulated sSTm and 2 was found in group F vs group A, *p* < 0.001. Likewise, the sST scores were considerably higher in the healthy group (sST1 18.56 ± 1.79; sST2 26.85 ± 2.81) than in the other, especially group F (sST1 11 ± 1.79; sST2 14.5 ± 2.38), *p* < 0.001 (Table 2). Statistical analysis revealed significant differences regarding the results of the sST between the groups, *p* < 0.001. Table 3 shows number of patients who obtained individual Schirmer test values—divided into 4 or 5 categories to facilitate the display of the results.

**Table 3:** Schirmer test results in the studied groups

Test	(mm)	Groups						Chi-squared	
		A	B	C	D	E	F		
								Value	*p*
Lacrimal Schirmer test	0–5	0	0	0	0	0	0	599.61	*p* < 0.001
	6–10	0	0	12	2	12	56		
	11–15	17	72	91	74	69	19		
	≥ 16	138	37	0	39	3	0		
Salivary Schirmer test mouth	0–5	0	0	0	0	0	0	679.91	*p* < 0.001
	6–10	0	1	16	1	12	28		
	11–15	9	86	84	58	68	46		
	16–20	129	22	3	56	4	0		
	≥ 21	18	0	0	0	0	0		
Salivary Schirmer test 2	0–5	0	0	0	0	0	0	654.49	*p* < 0.001
	6–10	0	0	2	0	2	6		
	11–15	0	9	44	0	23	41		
	16–20	2	40	60	37	50	16		
	≥ 21	154	60	1	78	9	0		

### Schirmer test correlations

There were positive correlations between the lST and the sST tests, especially in group F (lST vs sSTm *r* = 0.76, Schirmer test vs sSTp *r* = 0.76, *p* < 0.001) (Table 4). There was a strong correlation between sSTp and sSTm results in group F, *r* = 0.83, *p* < 0.001 (Table 5).

**Table 4:** Correlation between the lacrimal Schirmer test and salivary Schirmer tests 1 and 2

Groups		Salivary Schirmer test mouth			Salivary Schirmer test parotid		
		*N*	*r*	*p*	*N*	*r*	*p*
A	Lacrimal Schirmer test	156	0.53	*p* < 0.001	131	0.39	*p* < 0.001
B		109	0.46		97	0.41	
C		103	0.67		97	0.63	
D		115	0.67		106	0.65	
E		84	0.68		80	0.76	
F		75	0.76		65	0.76

**Table 5:** Correlation between the salivary Schirmer test mouth and parotid

Groups		Salivary Schirmer test mouth		
		*N*	*r*	*p*
A	Salivary Schirmer test parotid	131	0.49	*p* < 0.001
B		97	0.54	
C		97	0.86	
D		106	0.49	
E		80	0.65	
F		65	0.83	

## Discussion

This is one of the first studies in which lacrimal Schirmer test was adopted to measure salivary flow. Subjective complaints of dry mouth might not show salivary gland hypofunction, especially in non-Sjögren syndrome patients. Knowing the fact that submandibular and sublingual glands are main contributors to unstimulated whole saliva, we decided to locate Schirmer test in the floor of the mouth and in front of the parotic gland duct. Achieved results of those tests indicated strong relationship with lacrimal Schirmer test.

In our study, 424 patients had signs or symptoms of ocular and mouth dryness. Dry eye and concomitant xerostomia are not always associated with Sjögren syndrome, as they may occur in apparently healthy patients or those with systemic disorders. It is widely known that the prevalence of keratoconjunctivitis sicca increases with age, but age is not a major risk factor for salivary hypofunction [9]. Nevertheless, some epidemiological studies have demonstrated an upsurge in the prevalence and incidence of xerostomia with age [10]. There were statistically significant differences between the study groups as far as age was concerned. This was due to the fact that healthy patients were younger. Moreover, our analysis showed that elderly patients with systemic disease reported more subjective and objective symptoms than the healthy patients (*p* < 0.001).

Among our participants, there were more women than men (*p* = 0.043). This indicates a tendency for women to be more aware of their health and treatment; moreover, more women are affected by dry eye syndrome [11]. The higher incidence of reported problems in women than in men has also been confirmed [12]. This phenomenon has been described by many authors suggesting estrogen regulation of tear production may contribute to explain the female gender predilection in some ocular diseases, i.e., dry eye syndrome [13].

In our study, the eye Schirmer test values decreased with age and concomitant symptoms and general disease (*p* < 0.001). None of the patients from groups C or F achieved normal results from the eye Schirmer test (*p* < 0.001). These findings may suggest that systemic disease and occurrence of the subjective or objective symptoms of dry eye influence the test outcomes.

In 1948, Haessler used the Schirmer test by setting up the strip in the corner of the mouth on the inside of the lip. Then, in 1986, David and Marks performed the Schirmer tear strip to measure salivary flow rate near the parotid papilla for 5 min. The mean readings ranged from 10.6 to 15.6/3 min and 18–26 mm/5 min. Without comparing these results with other methods, they claimed that this was a quick and cost-effective tool [14]. Other authors obtained higher values of wetting the paper at the same time; for example, Lopez achieved mean values of 26 mm after 3 min and 43.3 mm after 5 min [15]. In our study, we observed mean values 20.33 mm/3 min. This contradiction may come from the different techniques of execution, the location of the strip, its slope to the mouth cavity, and possible factory differences in the physical properties of the paper test according to the companies.

Lopez-Jornet conducted a study on 159 healthy adult patients without any salivary problems measuring saliva flow rate by applying the Whatman paper strip under the tongue in contact with the mucosa and with the draining and the swab technique. Very weak interconnection (*r* < 0.3, *p* = 0.0002) was achieved by correlating the results with volumetric measurements. Despite this, the authors suggest using this oral equivalent of Schirmer test due to its ease-to-perform, inexpensiveness, and wide patient acceptability [16]. A few years later, the same author performed the oral Schirmer tests (unstimulated and acid-stimulated with the Whatman paper strip) and sialometric drainage tests. He received the following results: in the control group, the mean saliva flow was 40.92 ± 22.28 mm/5 min and 27.25 ± 24.11 mm/5 min in patients with SS and 36.84 ± 23.4 mm/5 min in patients with dry mouth. Based on their data, they suggested a cutoff ≤ 30 mm/5 min (67.9% sensitivity and 62.8% specificity). His study confirmed that the oral Schirmer test might be a good tool to diagnose xerostomia [16].

Later, many authors tried to find cutoff value for the unstimulated saliva Schirmer test with high sensitivity and specificity. According to Fontana, the modified Schirmer test value < 25 mm at 3 min suggests hyposalivation with high sensitivity (77%) and specificity (80%). Moreover, her study revealed moderate Spearman correlation coefficients (0.67–0.71) between the sST and the volumetric/gravimetric methods. In conclusion, this study supports use of the sST test as a screening tool for hyposalivation [17]. Löfgren et al. reported that a cutoff value ≤ 30 mm/5 min of the unstimulated oral Schirmer test had a sensitivity of 67.9% and a specificity of 62.8% [18]. Other researchers showed that the sST value > 28 mm at 3 min was normal. Chen et al. pointed out that the sST value < 15 mm at 3 min suggested severe xerostomia and hyposalivation of patients after head or neck radiotherapy. Chen suggested that the salivary Schirmer test was able to distinguish between healthy adult volunteers and subjects who experienced profound xerostomia and hyposalivation [19].

In our study, we observed a mean value of the sSTm— 18.56 mm/1 min in healthy group A, in comparison to group F, where the mean value of sSTm was 11 mm/1 min, *p* < 0.00001. There were significant differences between the lacrimal and salivary Schirmer tests and the occurrence of systemic disorders in the studied groups (in group A: sST1—18.56 ± 1.79 s, sSTp—26.85 ± 2.81 s, Schirmer test 18.61 ± 2.23 s vs group F: 11.00 ± 1.19 s, and 9.55 ± 1.29 s, *p* < 0.000001). We suggest performing the sST for 3 min; after that time, the wetness of the strip may exceed its length (30–35 mm). It could happen due to the higher secretion of saliva than tears. In addition, opening the mouth and the tongue lifting for a few minutes is a stimulation that leads to excessive saliva secretion. Thus, 3 min is the ideal period of time over which to perform sST.

The salivary Schirmer test has not been standardized yet. There is still a need for more precise studies on this test to determine the normal range and the cutoff value. Therefore, we should be aware of its limitations and carefully formulate conclusions. In light of these limitations, our study did the salivary Schirmer test in two locations within the oral cavity. The results from the bottom of the mouth were read after 1 and from the parotid area after 3 min. It is important to note that the description of the salivary Schirmer test in the parotid area was not found in available literature. This test is more difficult; this area complicates keeping the test strip in the correct position without touching the mucosa. Moreover, we observed much faster secretion of the saliva in this location. Sometimes, complete wetness of the strip resulted in a rejection of the test. The results were read after 3 min due to limited access and the fact that an attempt to get the result requires removing the strip and ending the test. We observed a very strong correlation between the outcomes between those two tests sSTm and sSTp (*r ϵ*(0.72–0.87), *p* = 0.000001). Our study confirmed the linear relationship between the lacrimal Schirmer test and salivary ST. We observed a lower correlation between those tests in groups A and B. Thus, there could be locally unknown factors affecting the outcomes of the tests in generally healthy patients.

There are limitations of our study; we did not perform any comparison with other sialometric method but, based on the literature, the sST showed an excellent correlation of 0.85 with spitting methods [20]. Moreover, we were not comparing lST and sST with tear meniscus height or staining scores. We also did not compare established tests for the measurement of stimulated and unstimulated salivary flow using Schirmer test.

This is the first study measuring the Schirmer test results in the eye and mouth simultaneously, performed in non-Sjögren symptomatic patients and asymptomatic healthy individuals. Because the glandular dysfunction which manifests as dry eyes and mouth does not only occur in Sjögren’s syndrome, it is valuable to conduct the research in another study group. Moreover, it is crucial to diagnose dry mouth objectively; there is still a need for a relevant test that measures the moisture of the oral cavity. Advantages of the MST over the traditional methods are simplicity, time-saving, ease-to-perform, and wide patient acceptability. Further studies are required to confirm our findings and standardize these tests.

## Conclusion

This is the first report comparing the Schirmer test results in eye and mouth simultaneously performed in non-Sjögren symptomatic patients vs asymptomatic healthy individuals. Results of this study reveal an excellent correlation between the eye Schirmer test and the salivary Schirmer tests. Thus, those tests may be routinely used as a screening tool to evaluate dry eye and xerostomia in non-Sjögren patients in everyday clinical practice.
